# Root of *Polygonum cuspidatum* extract reduces progression of diabetes-induced mesangial cell dysfunction via inhibition of platelet-derived growth factor-BB (PDGF-BB) and interaction with its receptor in streptozotocin-induced diabetic rats

**DOI:** 10.1186/1472-6882-14-477

**Published:** 2014-12-11

**Authors:** Eunjin Sohn, Junghyun Kim, Chan-Sik Kim, Kyuhyung Jo, Yun Mi Lee, Jin Sook Kim

**Affiliations:** Korean Medicine Based Herbal Drug Development Group, Herbal Medicine Research Division, Korea Institute of Oriental Medicine (KIOM), 1672 Yusengdaero, Yuseong-gu, Daejeon, 305-811 South Korea

**Keywords:** Diabetic nephropathy, Glomerular proliferation, *Polygonum cuspidatum*

## Abstract

**Background:**

Platelet-derived growth factor–BB (PDGF-BB) is highly expressed in the renal tissues of patients with diabetic nephropathy, and it plays an important role in the initiation and progression of diabetic nephropathy. The aim of this study was to evaluate the protective effects of root of *Polygonum* cuspidatum extract (PCE) on early renal glomerular proliferation in streptozotocin (STZ)-induced diabetic rats.

**Methods:**

PCE (100, 350 mg/kg/day) was administered to diabetic rats for 16 weeks. Blood glucose and albuminuria were measured. Renal histology, α-smooth muscle actin (α-SMA), and proliferating cell nuclear antigen (PCNA) expression levels were also examined.

**Results:**

After 16 weeks of treatment with PCE, severe hyperglycemia and albuminuria were observed in the diabetic rats. The expressions levels of α-SMA and PCNA proteins were significantly increased in the glomeruli of the diabetic rats. The expression levels of PDGF-BB and its receptor expressions were greatly increased in the glomeruli of the diabetic rats. However, PCE markedly reduced albuminuria in the diabetic rats. PCE inhibited α-SMA and PCNA up-regulation and ameliorated PDGF-BB and PEGFR-ß protein expression in the diabetic rats. In addition, the binding of PDGF-BB/PDGFR-ß was inhibited by PCE as shown by an *in vitro* assay.

**Conclusions:**

These results suggest that PCE has an inhibitory effect on mesangial proliferation in diabetic renal tissues via the inhibition of the interaction of PDGF-BB with its receptor. PCE may have beneficial effects in preventing the progression of diabetic nephropathy.

**Electronic supplementary material:**

The online version of this article (doi:10.1186/1472-6882-14-477) contains supplementary material, which is available to authorized users.

## Background

Diabetic nephropathy causes the majority of case of end-stage renal disease, and it is an important cause of increased mortality in patients with diabetes mellitus (DM) worldwide. Platelet-derived growth factor (PDGF) has been suggested to play a role in the pathophysiology of various fibroproliferative diseases, including pulmonary fibrosis, systemic sclerosis, liver cirrhosis, cardiovascular disease and kidney fibrosis [1, 2]. The up-regulation of the PDGF pathway has been shown in the kidneys of diabetic animals and patients with DM [2–4]. PDGF potently increases cell proliferation and matrix synthesis. PDGF-BB, which is an isoform of PDGF that has been the most closely linked with the development of various renal diseases, is produced by glomerular mesangial cells [5]. Moreover, PDGF-BB is highly expressed in diabetic renal tissues and plays important role in the initiation and progression of diabetic nephropathy [6]. Hyperglycemia has been is well characterized and albuminuria is considered to be one of the most sensitive markers of renal injury, and albuminuria significantly increased within 6–8 weeks in the streptozotocin (STZ)-induced diabetic animal model [7–10]. Moreover, the expression of the PDGF-B chain or PDGFR-ß in mesangial cells is actually increased in the glomeruli in this animal model [2].

Medicinal plants have been used as traditional remedies for hundreds of years. *Polygonum cuspidatum (P. cupidatum)* is the dried root of *Polygonum cuspidatum* Sieb. et Zucc. (Polygonaceae). It has been widely used as a medicinal herb for a variety of purposes in Asian countries, such as the treatment of inflammatory diseases, hepatitis, tumors, and diarrhea [11]. This herb has also been used to control oral disease in Korea [12]. In traditional Chinese medicine, *P. cuspidatum* has been used for its anti-diabetic, antibacterial properties [13–15]. Recently, it has been reported that an herbal formulation containing of *P. cuspidatum* prevented hepatic fibrosis by decreasing the expression of transforming growth factor-beta (TGF-ß) and α-smooth muscle actin (α-SMA) [16]. The general understanding of the pathogenic factors leading to renal fibrosis in diabetic nephropathy patients has considerably expanded, and the mechanisms involving TGF-ß and α-SMA have been found to be important to the development of diabetic renal fibrosis [17–19]. Therefore, in this study, we investigated the protective effect of an ethanol extract of *P. cuspidatum* (PCE) against renal injury and fibrosis in STZ-induced diabetic rats.

## Methods

### Preparation of *Polygonum cuspidatium*extract (PCE)

Root of *Polygonum cuspidatum* was purchased from a commercial supplier in Jung-dong, Daejeon, Korea, in November 2008 and identified by Prof. Ju Han Kim at the Department of Life Sciences, Gachon University. A voucher specimen was deposited at the Herbarium of the Diabetic Complication Research Team, Korea Institute of Oriental Medicine (KIOM). The dried and ground plant material (6.8 kg) was extracted with ethanol (3 × 36 L) by maceration at room temperature for 3 days, and the extracts were combined and concentrated *in vacuo* at 40°C to create lyophilized PCE (580 g).

### Animals and induction of diabetes

The experiments were performed in accordance with the National Institutes of Health (NIH) Guide for the Care and Use of Laboratory Animals and approved by the Korea Institute of Oriental Medicine Institutional Animal Care and Use Committee. In our study, six-week-old male SD rats purchased from the Charles River Laboratory (Waltham, MA, USA) were acclimated for 1 week prior to the study. Our study was initiated using 8-week-old male SD rats (weighting, ~200 g), which were monitored for 16 weeks. Diabetes was induced in the rats by a single injection of streptozotocin (STZ, 60 mg/kg, i.p.). Age-matched control rats received an equal volume of vehicle (0.01 M citrate buffer, pH 4.5). Two days after the injection, a blood sample was collected from the ratil vein to measure the blood glucose level. Rats with a blood glucose level over 300 mg/dl were considered diabetic rats. At 8 weeks of age, the rats were randomly assigned to one of four groups (n = 8). PCE was dissolved in vehicle (0.5% w/v carboxyl methylcellulose solution) to a concentration of 50 mg/ml. Two groups of STZ-induced diabetic rats received daily gastric gavage of PCE (100 and 350 mg/kg), and the other groups were administered the same amount of vehicle gavage for 16 weeks. Blood glucose levels and body weight were monitored consecutively.

### Metabolic and morphological analyses

After 16 weeks of treatment, blood glucose was measured using an automated analyzer (Hitachi, Tokyo, Japan). Blood samples were collected from the tail vein after a 16-h fast. Individual rats were placed in metabolic cages to obtain 24-h urine collections, and urinary albumin excretion levels were measured using a sandwich enzyme-linked immunosorbent ELISA assay kit according to the manufacturer’s manual (Life Diagnostics, Inc., PA, USA). Rat albumin present in the urine sample was captured by an anti-rat albumin antibody that had been pre-adsorbed on the surfaces of microtiter wells. After sample binding, unbound proteins and molecules were washed off with washing buffer, and a biotinylated detection antibody was added to the wells to bind to the captured albumin. Strepavidin-conjugated horseradish peroxidase (SA-HRP) was then added to catalyze a colorimetric reaction with the chromogenic substrate 3,3′,5,5′-tetramethylbenzidine(TMB). This colorimetric reaction produces a blue product, which turns yellow when the reaction is terminated by the addition of stop solution (0.1 M H_2_SO_4_). The resulting, yellow reaction products were read at 450 nm using a microtiter plate reader (Bio-Tek, Winooski, VT, USA). Renal cortexes were fixed in 10% formaldehyde and embedded in paraffin, and 4-μm-thick sections were prepared. The sections were stained with periodic acid-Schiff (PAS) reagent and hematoxylin as a counterstain.

### Western blot analysis

The renal cortex was lysed with RIPA buffer (pH 7.5) (Pierce, IL, USA). Western blot analysis was performed as previously described [20]. Equal amounts (30 μg) of protein were separated by electrophoresis on SDS-polyacrylamide gels, electroblotted on polyvinylidene fluoride (PVDF) membranes and probed using primary anti-PDGF-BB and anti-PDGFR-ß (R&D Systems, MN, USA). The immunoblots were developed using an enhanced chemiluminescence detection system (Amersham Bioscience, NJ, USA) with HRP-conjugated secondary antibodies. Protein expression levels were determined by analyzing the signals captured on the PVDF membranes using an image analyzer (Las-3000, Fuji Photo, Tokyo, Japan).

### Immunohistochemical and immunofluorescent staining

Renal cortexes were fixed in 10% formaldehyde and embedded in paraffin, and 4-μm-thick sections were prepared. Staining was performed as previously described [15]. The antibodies used included rabbit anti-proliferating cell nuclear antigen (PCNA, Santa Cruz, CA, USA), mouse anti-PDGF-BB (R&D Systems, MN, USA), mouse anti- PDGFR-ß (R&D Systems), rabbit anti-α-SMA (Santa Cruz, CA, USA) and mouse anti-thy 1.1 (a mesangial cell marker; Millipore, MA, USA). For the detection of PDGF-BB and PDGFR-ß, the sections were incubated using an Envision kit (DAKO, CA, USA) and visualized by 3,3′-diaminobenzidine tetrahydrochloride (DAB). To detect PCNA and α-SMA, the sections were incubated with Texas red-conjugated goat anti-rabbit IgG (Santa Cruz, CA, USA) and fluorescein isothiocyanate-conjugated goat anti-rabbit IgG (Santa Cruz, CA, USA), respectively, and detected by fluorescence microscopy (Olympus, Tokyo, Japan). To characterize the proliferation of the mesangial cells, we performed double staining for PCNA and Thy 1.1. using the same section. To prevent cross-reactions between the two labeling procedures, the slides were incubated with normal mouse serum (Dako, CA, USA). To detect PCNA and Thy 1.1, the sections were incubated with fluorescein isothiocyanate-conjugated goat anti-rabbit IgG (Santa Cruz, CA, USA) and Texas red-conjugated goat anti-mouse IgG (Santa Cruz, CA, USA), respectively. Negative controls for immunohistochemistry were prepared by incubating the sections with nonimmune serum instead of primary antibody. For morphometric analysis, the number of positive cells or positive signal areas per one glomerulus in a total of 50 glomeruli was determined using ImageJ software (NIH, Bethesda, MD, USA).

### *In vitro*assay for ligand receptor binding inhibition

To investigate the inhibitory effect of PCE on PDGF-BB/ PDGFR-ß ligand receptor binding, ligand binding was examined by a sandwich ELISA assay *in vitro*. A total of 50 μl of a 0.05 μg dose of recombinant human PDGF-BB (R&D Systems, MN, USA) was pre-coated and incubated on a microplate for 24-h at 4°C and was then washed with PBS (pH 7.4). Next, the sample was blocked with 100 μl of CAS block solution (Life Technologies, CA, USA) for 30 min at 37°C and then washed with PBS. The sample was then added to 50 μl recombinant human (rh) PDGFR-ß /FC chimera labeled with peroxidase (Dojindo, Kumanoto, Japan) and 50 μl of a serial dilution of the PCE mixture in a microplate pre-coated with rh PDGF-BB. The samples were incubated for 1 h at 37°C and then washed with PBS. A TMB chromogen solution was used as a substrate for the horseradish peroxidase. After the reaction was terminated with stop buffer (0.1 M H_2_SO_4_), and a yellow reaction product formed, its absorbance was measured at 450 nm using a microtiter plate reader (Bio-Tek, Winooski, VT, USA). The inhibition of PDGF-BB ligand receptor binding was expressed as the percentage decrease in optical density (OD 450 nm). We calculated the IC_50_ concentration (ug/ml) as the 50% inhibition of PDGF-BB/ PDGFR-ß ligand binding.

### Statistical analysis

The data are expressed as the mean ± SEM and were analyzed by one-way analysis of variance (ANOVA), followed by Tukey’s multiple comparison test or by an unpaired Student’s *t-*test using GraphPad *Prism 5.0* software (GraphPad, San Diego, CA, USA). Differences of P < 0.01 were considered statistically significant.

## Results

### Body weights and metabolic parameters

At 16 weeks of treatment, the body weights were significantly decreased in the vehicle-treated diabetic rats compared with the normal control rats. However, there was no difference in body weight between the vehicle-treated diabetic rats and PCE-treated diabetic rats. The blood glucose levels increased by approximately 3-fold in the vehicle-treated diabetic rats compared with normal control rats (P < 0.01). There was no statistically significant difference between the vehicle-treated diabetic rats and PCE-treated diabetic rats with respect to blood glucose levels (Table 1). These resulting data demonstrated that PCE maybe has independent effect on glucose-lowering and body weight.

**Table 1:** **Metabolic and physical parameters**

	NOR	DM	PCE-100	PCE-350
Blood glucose (mg/dl)	162.1 ± 11.0	587.3 ± 43.5*	530.3 ± 58.1	474.2 ± 88.4
Body weight (g)	475.9 ± 10.7	208.5 ± 12.1*	205.5 ± 23.1	206.0 ± 16.4

NOR, normal rat; DM, STZ-induced diabetic rat; PCE-100, DM treated with PCE (100 mg/kg); PCE-350, DM treated with PCE (350 mg/kg). All data are expressed as the mean ± SEM (n=8). *P <0.01 vs. NOR group.

### Morphology and urinary albumin levels

**Figure 1:**
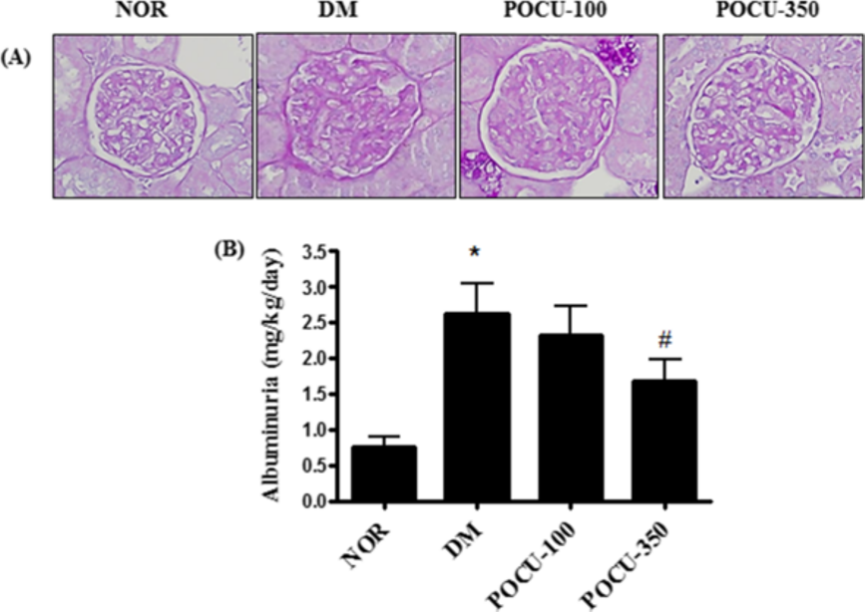
**Renal histopathology and albuminuria. (A)** Periodic acid-Schiff staining of glomeruli. 400× magnification. **(B)** Albuminuria in the experimental group. NOR, normal rat; DM, STZ-induced diabetic rat; PCE-100, DM treated with PCE (100 mg/kg); PCE-350, DM treated with PCE (350 mg/kg). All data are expressed as the mean ± SEM (n=8). *P <0.01 vs. NOR group, # P <0.01 vs. DM group, and +P <0.01 vs. PCE-100 group.

Mesangial matrix expansion is considered a hallmark of diabetic nephropathy [21]. At 24 weeks of age, vehicle-treated diabetic rats showed focal mesangial matrix expansion, and albuminuria was significantly increased in the vehicle-treated diabetic rats compared to the normal control rats (Figure 1A). However, PCE treatment ameliorated the mesangial expansion and albuminuria compared with the vehicle-treated diabetic rats (Figure 1A and B).

### PDGF expression in renal glomeruli

**Figure 2:**
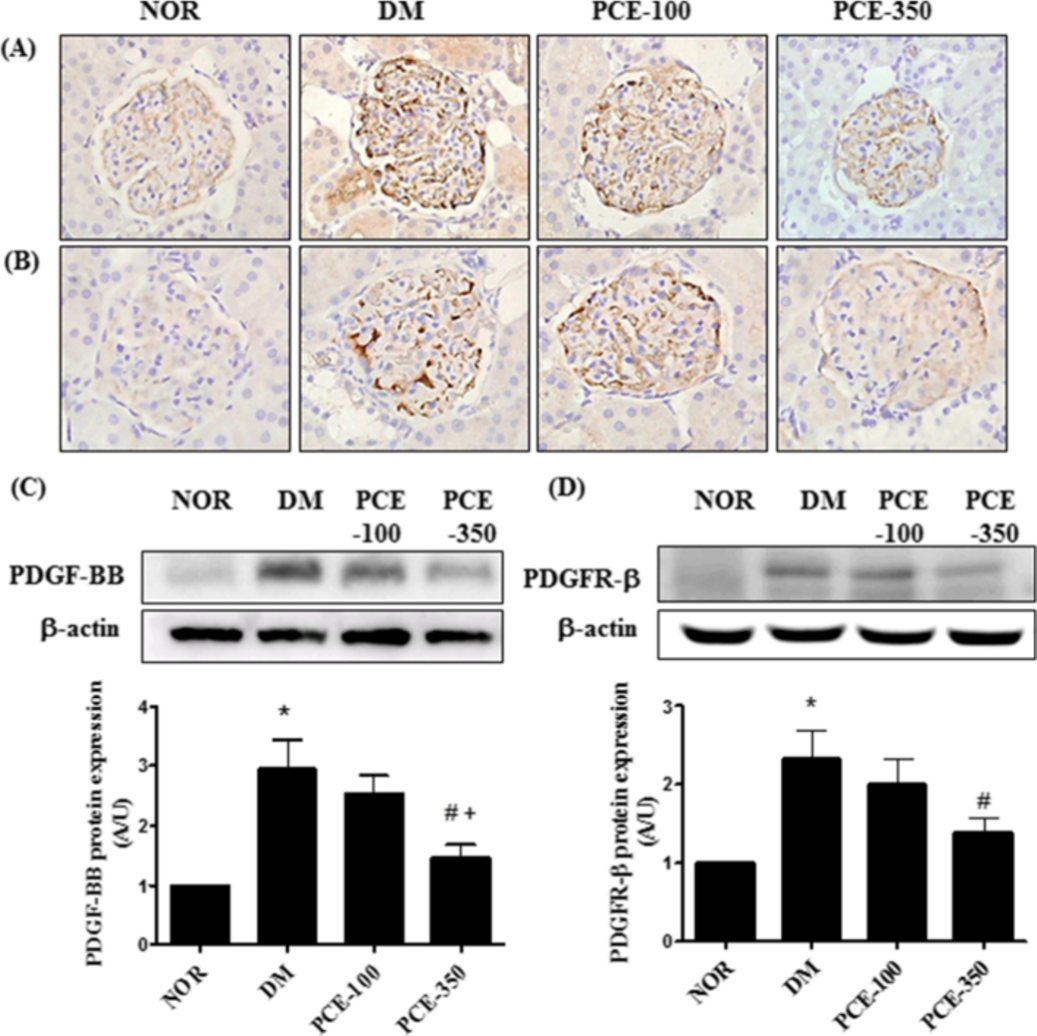
**Effects of PCE on PDGF-BB and PDGFR-ß in the renal glomeruli. (A)** Immunohistochemical staining of PDGF-BB and **(B)** PDGFR-ß. 400× magnification. Morphometric analysis of western blots showing **(C)** PDGF-BB and **(D)** PDGFR-ß expression in the renal cortex for each group. All data are expressed as the mean ± SEM (n=8). *P <0.01 vs. NOR group; #P <0.01 vs. DM group, and +P <0.01 vs. PCE-100 group.

To investigate the effect of PCE on expression of PDGF and its receptor in diabetic renal injury, immunohistochemistry and western blotting analysis were performed. Immunohistochemical staining of PDGF-BB and PDGFR-ß in the glomeruli were mainly localized to the glomerular mesangial cells revealing a significant increase in the vehicle-treated diabetic rats compared with the normal control rats. This increase of PDGF-BB and PDGFR-ß expression in the glomeruli in the vehicle-treated diabetic rat was attenuated by PCE treatment (Figure 2A and B). Western blot analysis showed that, PDGF-BB and its receptor in the renal cortex were also markedly increased in the vehicle-treated diabetic rats compared with the normal control rats. PCE reduced these diabetes-induced increases in PDGF-BB and PDGFR-ß expressions in a dose-dependent manner (Figure 2C and D). This result demonstrated that PCE prevents renal mesangial proliferation in diabetic nephropathy.

### Inhibition of PDGF-BB ligand receptor binding assay

**Figure 3:**
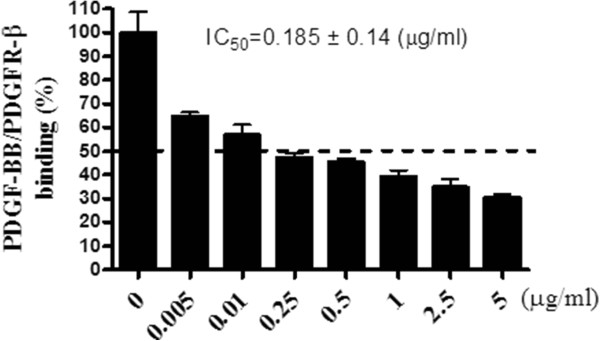
**Inhibitory effects of PCE on the binding of the PDGF-BB ligand with its receptor** ***in vitro*** **.** The experiments were performed in triplicate. All data are expressed as the mean ± SEM.

To investigate whether PCE could inhibit the binding of the PDGF ligand to its receptor, a sandwich ELISA assay was performed. PCE showed inhibition effect on binding of the PDGF ligand to its receptor in a concentration-dependent manner. PCE exhibited an inhibitory effect on the binding of the PDGF-BB ligand to its receptor (IC_50_ = 0.185 ± 0.14 μg/ml) (Figure 3).

### Expression of α-SMA and PCNA in renal glomeruli

**Figure 4:**
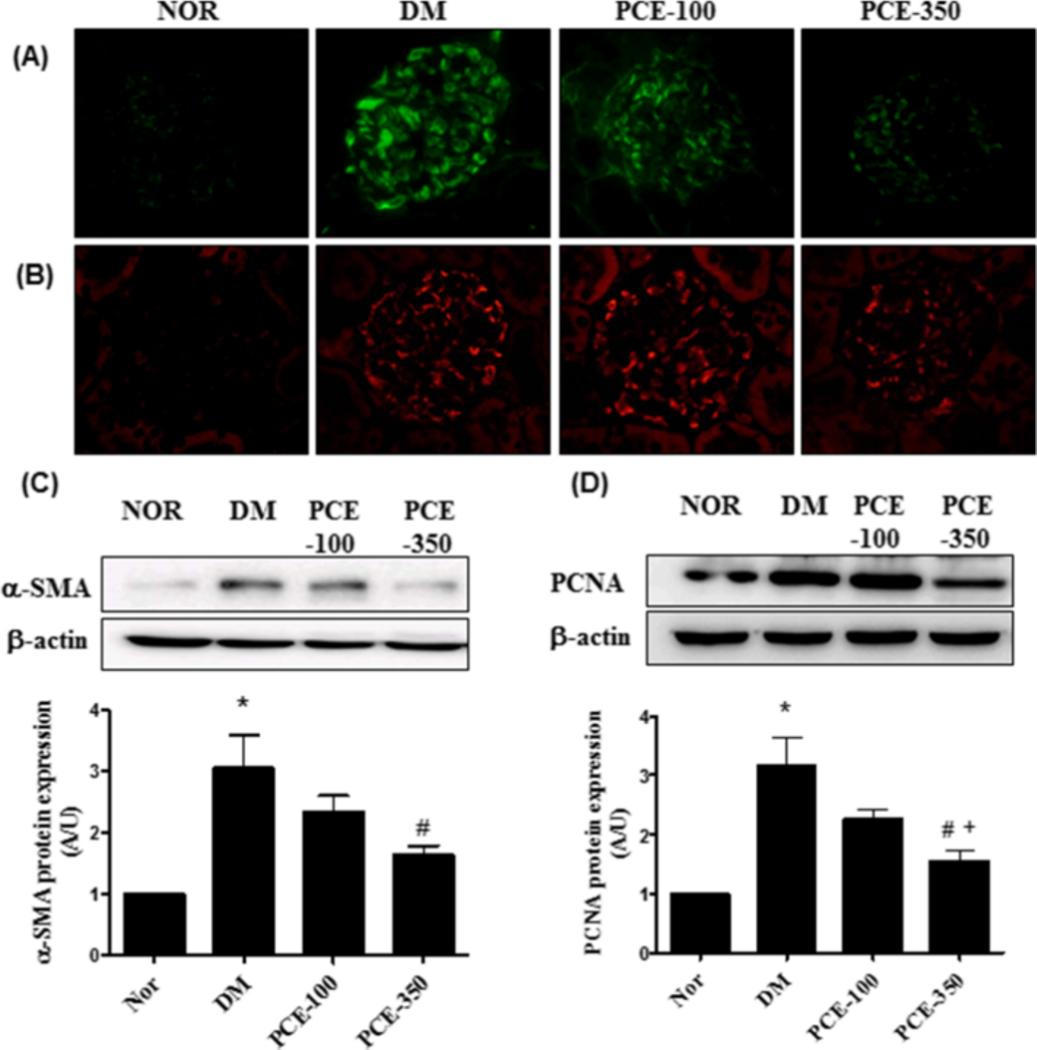
**Effect of PCE on α-SMA and PCNA expression in the renal glomeruli. (A)** Immunofluorescence staining for α-SMA and **(B)** PCNA 400× magnification. Western blot analysis of **(C)** α-SMA and **(D)** PCNA expression in the renal cortex for each group. All data are expressed as the mean ± SEM (n=8). *P <0.01 vs. NOR group; #P <0.01 vs. DM group, and +P <0.01 vs. PCE-100 group.

**Figure 5:**
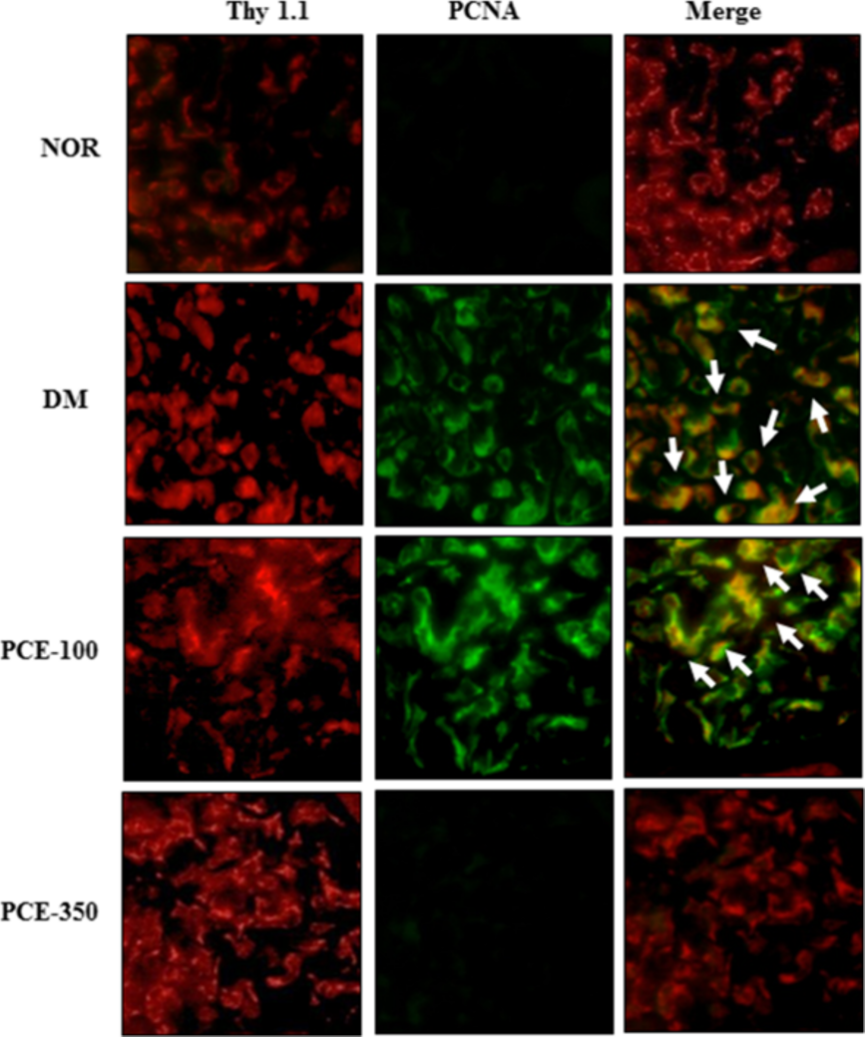
**Effect of PCE on mesangial cell proliferation in the renal glomeruli.** Immunofluorescence staining was performed with anti-thy 1.1, which is a specific marker for mesangial cells, and anti-PCNA antibody and visualized with Texas-red and fluorescein isothiocyanate (FITC), respectively, using a fluorescence microscope. Representative merged micrographs (yellow) of four independent experiments are shown at 400× magnification.

Mesangial proliferation is considered a prominent feature of diabetic nephropathy. The expression level of proliferation markers, such as α-SMA and PCNA, were measured using immunohistochemistry and western blot analysis. Immunohistochemical staining of α-SMA and PCNA was markedly more visible in the diabetic renal glomeruli compared with normal glomeruli. PCE treatment showed decreased expression of α-SMA and PCNA in the diabetic glomeruli (Figure 4A and B). The western blot assay indicated that, the expressions levels of α-SMA and PCNA were significantly increased in the vehicle-treated diabetic rats compared with the normal control rats. The high-dose PCE treatment markedly reduced these diabetes-induced increases in α-SMA and PCNA expression (Figure 4C and D). In addition, to investigate whether the enhanced protein expression was colocalized to the mesangial cells of the glomeruli, PCNA and Thy 1.1, which is a mesangial cell marker [22], were visualized by double immunofluorescence staining. Thy 1.1 was positively stained in the glomerular mesangial cells for all of the experimental rats. PCNA expression was significantly increased in the vehicle-treated diabetic rats compared with the normal control rats. In the dual-labeled section, PCNA expression was localized to a region of Thy 1.1-positive mesangial cells in the diabetic renal tissue (Figure 5). However, the high-dose PCE treatment significantly decreased PCNA expression in the diabetic rats (Figure 5).

## Discussion

Our current study demonstrated that PCE treatment significantly improved albuminuria, which is a biochemical marker of renal function in diabetic rats. In addition, PCE prevented mesangial expansion and proliferation in these diabetic rats. Of particular interest, our study showed that PCE exhibits inhibitory activity against the binding of the PDGF-BB ligand to its receptor, PDGFR-ß. Moreover, PCE reduced the expression of α-SMA and PCNA in the diabetic rats.

Hyperglycemia and albuminuria are present in STZ-induced diabetic rats, and diabetic nephropathy progresses rapidly in this animal model [23]. Hyperglycemia leads to the destruction of the glomerular filtration barrier, leading to glomerular damage and resulting in urinary protein or albumin leakage, exacerbating the progression of diabetic nephropathy [24, 25]. Albuminuria is the main pathologic feature of many primary glomerular diseases including diabetic nephropathy [7]. Consistent with this interpretation, our study demonstrated that albuminuria and focal glomerular matrix expansion were markedly increased in the vehicle-treated diabetic rats. However, our study showed that PCE treatment ameliorated the enhanced diabetes-induced renal dysfunction, such as albuminuria glomerular matrix expansion. Although *P. cuspidatum* has anti-diabetic property [13], our data shows that PCE inactived on blood glucose and body weight in diabetic induced rat. However, various anti-diabetic drugs, such as metformin, dipeptidyl peptidase 4 (DPP4) inhibitors, although it has been successfully work to blood glucose lowering in type 2 diabetes, have been repurposed from other clinical indications to treat renal injury. Actually, Kanasaki et al. and Liu et al. investigated the anti-fibrotic effect of linagliptin, DPP4 inhibitor, in type 1 model of diabetic nephropathy [26, 27]. These studies demonstrate that an insulin-deficient model of diabetes enable the evaluation of the effects of the DPP4 inhibitor independent of glycemic control and body weight. Also, these studies provide information regarding renal benefit of DPP4 inhibitor independent of glycemic control and body weight. Moreover, metformin, a well-known anti-diabetic drug for type 2 diabetes mellitus (T2DM), had no effect on body weight and blood glucose in type 1 diabetes mellitus (T1DM) [28, 29]*.* However, our study demonstrated that PCE has effect on the reno-protective action. Therefore, PCE possibly has reno-protective effect independent body weight and hypoglycemic effect.

In this study, we also found that the treatment of diabetic rats with PCE ameliorated mesangial expansion by inhibiting the binding activity of PDGF-BB to its receptor, PDGFR-ß. Mesangial expansion is one of the major pathologic factors of diabetic nephropathy. The proliferation of mesangial cells is a prominent feature of glomerular diseases, including diabetic nephropathy [30]. PDGF has been consistently implicated in the cell proliferation and extracellular matrix (ECM) accumulation that characterizes progressive glomerular diseases [30, 31]. PDGF-BB is one of the classical isoforms of PDGF, which consists of two PDGF-B chains linked by a disulfide bond. Moreover, PDGF-BB has been demonstrated to play important roles in the initiation and progression of diabetic nephropathy [32]. PDGF-BB has received considerable attention as the major mediator of mesangial cell proliferation, promoting the up-regulation of PDGF and its receptor in mesangial cells [5, 33]. In the early stages of diabetic nephropathy, mesangial cells express high levels of PDGF-BB, which binds to PDGFR-ß, resulting in an increase glomerular volume, inducing the proliferation and overgrowth of mesangial cells [5]. The up-regulation of PDGF-BB and its receptor in the mesangial cells of diabetic rats may be responsible for the proliferation of these cells and the accumulation of ECM proteins in the mesangium as observed in the early stages of diabetes [2, 34]. In addition, ECM proteins, such as α-SMA and PCNA, have been regarded as appropriate indicators of mesangial proliferative glomerulonephritis, including diabetic nephropathy [35, 36].

*P. cuspidatum* has been broadly utilized as a medicinal herb in Asian countries, including Korea. In our study, PCE exhibited inhibitory activity on the binding of the PDGF-BB ligand to its receptor, PDGFR-ß, showing an IC_50_ value of 0.185±0.14 μg/ml. We also performed a dot blot assay to determine the affinity of PCE for PDGF-BB or PDGFR-ß (see Additional file 1). This assay showed that PCE did not directly bind to PDGF-BB or PDGFR-ß, indicating its lack of selective affinity. Resveratrol and emodin, which are major components of the activated form of PCE, are phenolic compounds that are concentrated in *P. cuspidatum* and have been studied for their anti-diabetic effects *in vitro*[37, 38]. An ethanolic extract of *P. cuspidatum* has been demonstrated to exhibit comprehensive and suppressive effects on inflammatory and oxidative stresses [39] and significantly enhance the rate of wound healing in rats and humans [40, 41]. In our previous study, an extract of *P. cuspidatum* radix has been shown to exhibit strong anti-lipase activity [42]. Resveratrol is a natural anti-oxidant polyphenol that is present in *P. cuspidatum* and has received increased attention in recent years. This strong polyphenolic compound has been shown to have several biological functions, including anti-inflammatory and antioxidant activities [43]. Additionally, emodin has an inhibitory effect on high-glucose induced TGF- ß1 and fibronectin expression in cultured mesangial cells [44]. In our study, PCE showed potential for preventing mesangial proliferation and ECM overgrowth in the diabetic kidney due to its inhibitory effects on PDGF-BB/PDGFR-ß binding. Although the major chemical compounds of PCE include resveratrol and emodin, we did not assess the combined effect of these compounds on diabetic nephropathy. However, a previous study has demonstrated that resveratrol or/and emodin ameliorates renal function in diabetic rodents and suppresses high glucose-induced glomerular mesangial cell proliferation by inhibiting the NF-kB pathway [44–47]. Based on previous reports and our *in vivo* results, the ability of PCE to protect against renal damage may be due to the effect of these compounds.

## Conclusions

In conclusion, renal mesangial expansion and albuminuria in diabetic rats were ameliorated by treatment with PCE. PCE exerts anti-proliferative effects via inhibition of the PDGF-BB/PDGFR-ß signaling pathway in diabetic renal glomeruli. Therefore, our study indicates that therapies targeting mesangial proliferation may be significantly beneficial to patients with diabetic nephropathy

## Electronic supplementary material

Additional file 1: **Supporting Data.** (DOCX 42 KB)
