# Supplementary material for: Root of Polygonum cuspidatum extract reduces progression of diabetes-induced mesangial cell dysfunction via inhibition of platelet-derived growth factor-BB (PDGF-BB) and interaction with its receptor in streptozotocin-induced diabetic rats
Source: BMC Complement Altern Med. 2014 Dec 11;14:477. doi: 10.1186/1472-6882-14-477 (PMC4364577; doi:10.1186/1472-6882-14-477)

**Additional file 1.**

Methods

Dot binding assay

To demonstrate whether PDGF-BB or PDGFR-β directly interacts with PCE, we performed a dot binding assay. Recombinant human PDGF-BB or PDGFR-β, and PCE (1, 0.1, 0.01ug/ul) were directly spotted on the nitrocellulose (NC) membrane (Bio-Rad Laboratoties, MA, USA) due to a lesser extent of sample, repectively. The membrane was soaked in buffer (25 mM Tris, 192mM Glycine and 20% methanol) for 30 sec and then blocked with BSA (5% in PBS) for 30 min at RT. After brief washing with PBS, the membrane was incubated with PDGF-BB or PDGFR-β (0.5 ug /ml in PBS) for overnight at 4 ℃. After washing, the membrane was then incubated with anti-PDGF-BB or PDGFR-β antibody (2 ug ⁄ ml in 1% BSA-containing PBS) for 1 hr at RT, respectively. After another brief wash, the membrane was incubated with horseradish peroxidase-conjugated second antibody and then developed by enhanced chemiluminescence (ECL, Amersham Bioscience, NJ, USA). The appearance of spots on the NC membrane represents the direct interaction of PDGF-BB or PDGFR-β with PCE coated on the NC membrane.

Legends for figure

Figure1. Dot blot assay to determine affinity of PCE against PDGF-BB or PDGFR-β. Recombinant human PDGF-BB (A) or PDGFR-β (B) and PCE were immobilized on the nitrocellulose (NC) membrane. After incubation with PDGF-BB or PDGFR-β, the membrane was further incubated with antibodies against PDGF-BB or PDGFR-β, respectively, and then developed. We observed that immobilized PDGF-BB or PDGFR-β can be recognized by the anti- PDGF-BB or PDGFR-β, respectively. PCE did not directly bind to PDGF-BB or PDGFR-β. These results indicates that PCE has no affinity to PDGF-BB or PDGFR-β.


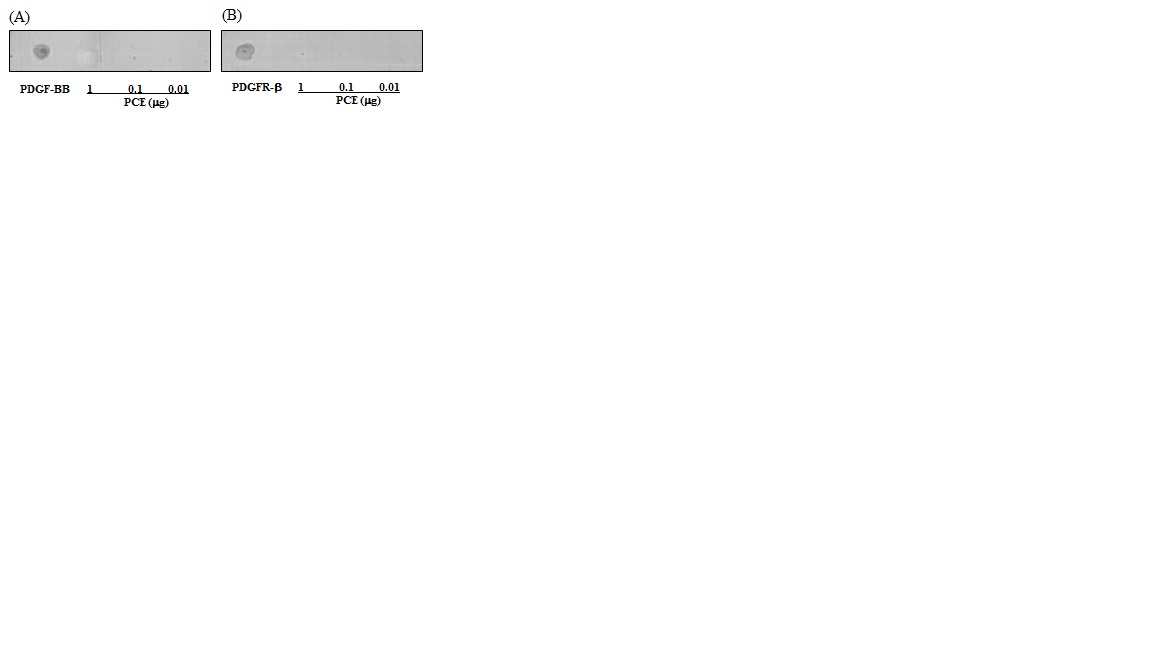

Supplement: Supplementary file 1 — Additional file 1: Supporting Data. (DOCX 42 KB) [file 12906_2014_2093_MOESM1_ESM.docx]
